# Supplementary material for: Synergistic Activity of Mobile Genetic Element Defences in Streptococcus pneumoniae
Source: Genes (Basel). 2019 Sep 13;10(9):707. doi: 10.3390/genes10090707 (PMC6771155; doi:10.3390/genes10090707)
Supplement: Supplementary file 1 [file genes-10-00707-s001.pdf]

Supplementary Material

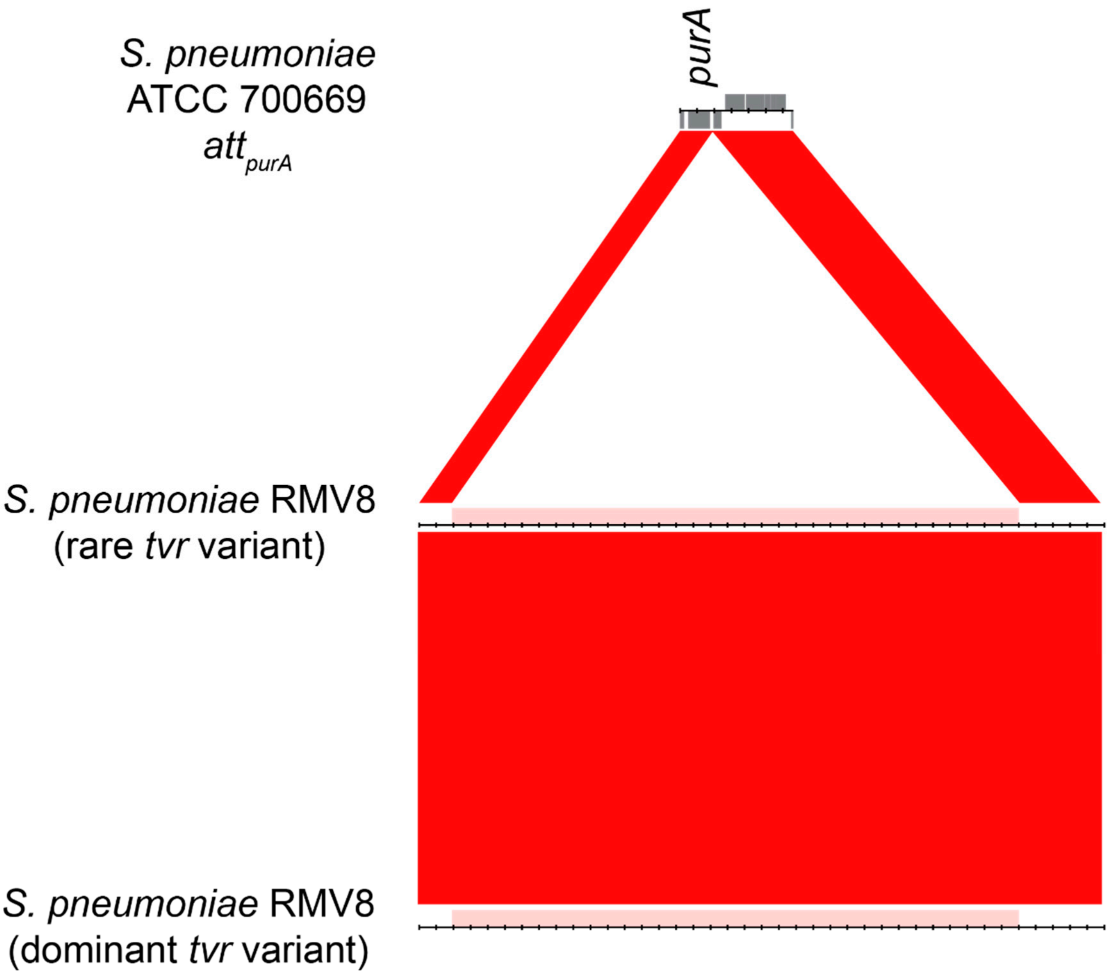

Figure S1. Vertical inheritance of mobile genetic elements in *S. pneumoniae* expressing different variants of the phase-variable SpnIV RMS. The alignment shows the empty att_{purA} prophage insertion site in *S. pneumoniae* ATCC 700669 above two variants of *S. pneumoniae* RMV8, which both carry a prophage inserted at this position, indicated by the pink bar annotated on each. Red bands linking the sequences indicate BLASTN matches; the tick marks on the sequences are present at a spacing of 1 kb. The prophage is retained in the two RMV8 genotypes (ENA accession codes ERS2478544 for the rare variant, and ERS1681527 for the dominant variant), which are descended from the same original culture, but express different variants of the *tvr* locus and have different methylation patterns.

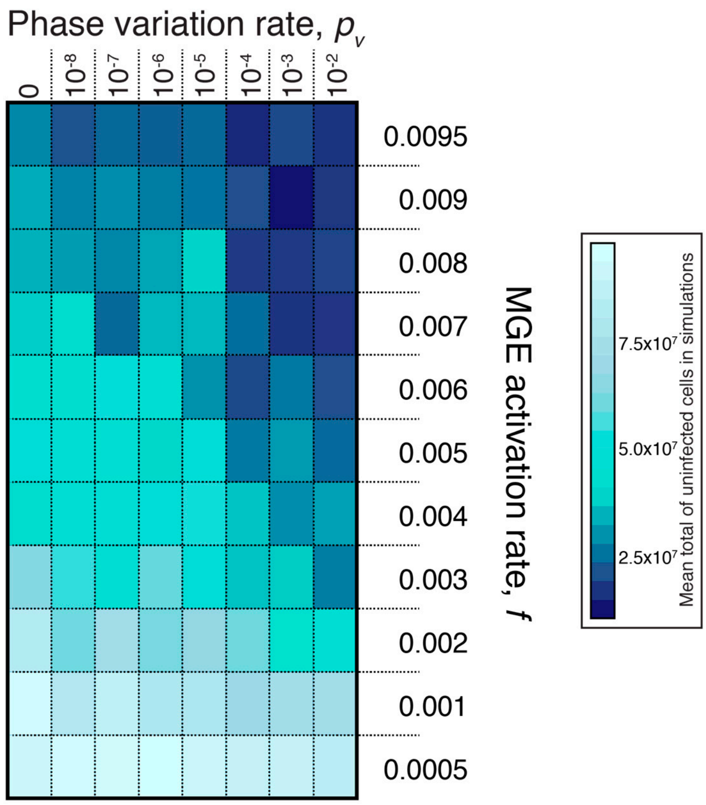

Figure S2. Heatmap displaying the results of simulations in which a cellular population expressing a phase-variable RMS was repeatedly challenged by invading MV-type MGEs. These simulations were identical to those analysed in Figure 1, except the MV-type MGEs spread more through vertical transmission, relative to horizontal transmission, when compared to ML, and correspondingly the rates of activation (*f*) could be lowered 100-fold. Each cell corresponds to a particular parameter combination, with p_v determined by the column, and the rate of MGE activation, *f*, determined by the row. The colour of the cell represents the mean number of uninfected cells that survived over the 10^4 timesteps of the simulation, across 20 replicates. Results are only shown for a small ($\kappa = 10^4$) cell population; to enable effective invasion of a cell population of this size, MV's β was raised to 5×10^{-3} t^{-1} and the invasion rate, m_i, was 5×10^{-7} t^{-1}.

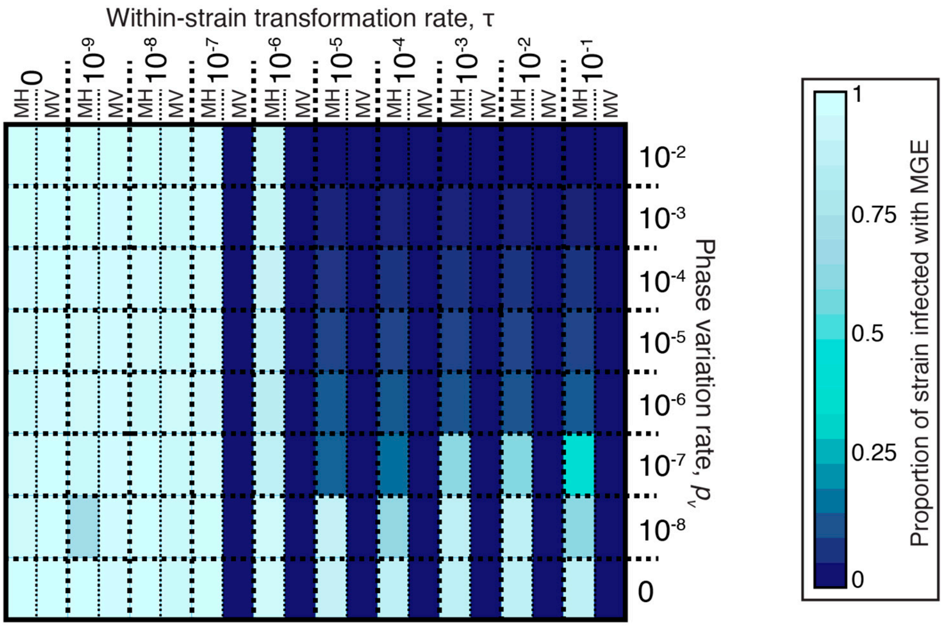

Figure S3. Heatmap showing the results of simulations of sequence exchange between a transformable *S. pneumoniae* population, initiated at carrying capacity, expressing a phase-variable RMS. The simulations differed from those presented in Figure 5, which were initiated from a small inoculum of 100 cells of each genotype, in that these cell populations were initiated at the carrying capacity of 10^6. The starting mix was a 99:1 ratio of uninfected cells of variant A, and infected cells of variant B. Variation in within-strain transformation rate (τ) is shown across columns, and variation in p_v shown across rows. Each cell is split in two, with the halves showing the proportion of cells infected with an MGE in simulations with MH (left) and MV (right). These values are calculated as the mean across three replicate simulations. The results are similar to those in Figure 5b, demonstrating they are not an artefact of the initial growth phase of the population.

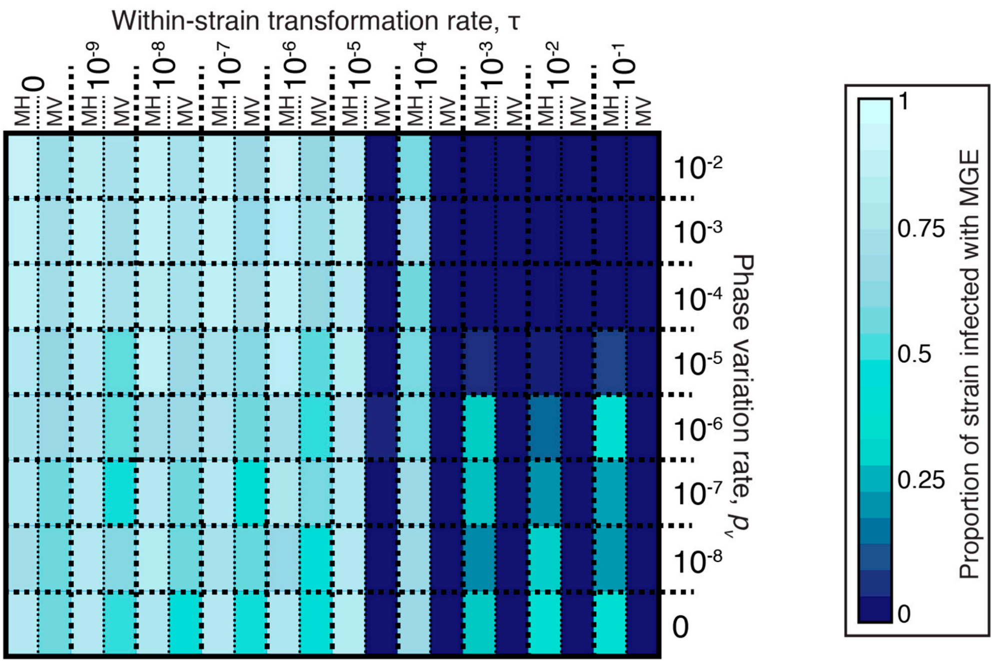

Figure S4. Heatmap showing the results of simulations of sequence exchange between a transformable *S. pneumoniae* population, initiated at carrying capacity, expressing a phase-variable RMS. Variation in within-strain transformation rate (τ) is shown across columns, and variation in p_v is shown across rows. The simulations differed from those presented in Figure 5, which were initiated from a small inoculum of 100 cells of each genotype, in that these cell populations were initiated at the carrying capacity of 10^4. The starting mix was a 99:1 ratio of cells of variant A and variant B, all uninfected. This cellular population was challenged by MGEs invading the population at a rate, m_i, of $5 \times 10^{-7}\ t^{-1}$, as for the simulations analysed in Figure S2. The invading MGE was either MV, with the modified β of $5 \times 10^{-3}\ t^{-1}$ as in Figure S2, or MH, with the modified β of $1 \times 10^{-4}\ t^{-1}$. Each cell is split in two, with the halves showing the proportion of cells infected with an MGE in simulations with MH (left) and MV (right). These values are calculated as the mean across three replicate simulations. The results are similar to those in Figure 5b, demonstrating they are not an artefact of the level at which the MGEs were originally seeded into the population.

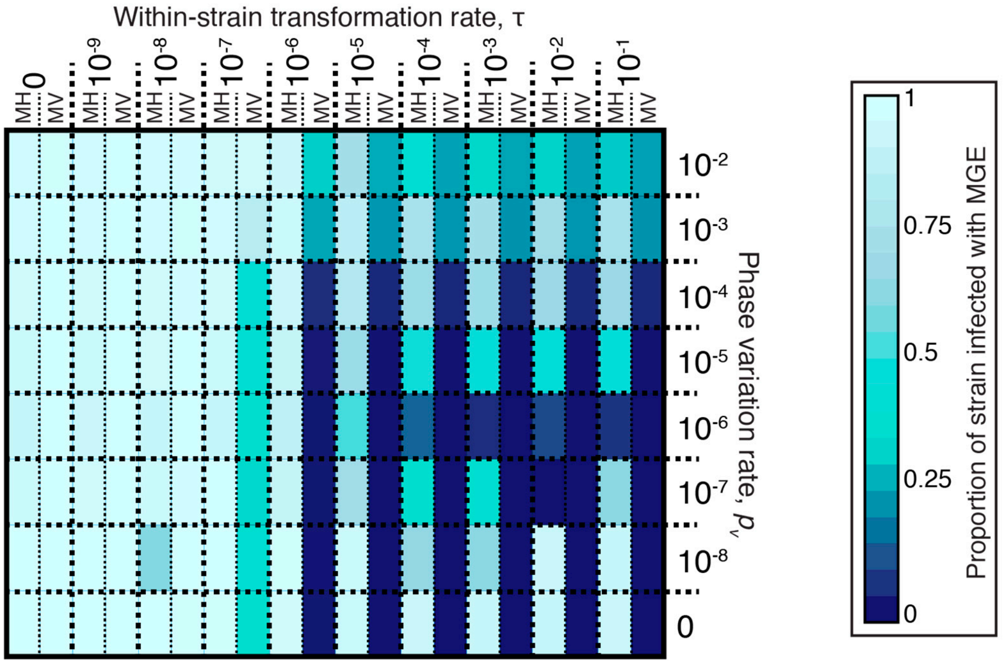

Figure S5. Heatmap showing the outputs of simulations of sequence exchange between a transformable *S. pneumoniae* population expressing a phase-variable RMS, in which variant B is 100-fold less transformable than variant A. Variation in within-strain transformation rate (τ) is shown across columns, and variation in p_v shown across rows. The annotated τ corresponds to the transformation rate of variant A; that of variant B is 0.01τ. Each cell is split in two, with the halves showing the proportion of cells infected with an MGE in simulations with MH (left) and MV (right). These values are calculated as the mean across three replicate simulations.

Table S1. Primers used to quantify alleles of the *tvr* locus in *S. pneumoniae* RMV5 and RMV8. The TRDII/IV_For primer was used as the forward primer in each reaction, with each reverse primer specific to a different variant of the *tvr* locus.

Primer name	Sequence
TRDII/IV_For	GCAAGTGAGCTGGATTTGTTAAG
TRDi_Rev	CTCCTCACTAAACAACTCATCTGA
TRDii_Rev	CTTCAAAGGAGGAAATCAGATGGTAG
TRDiii_Rev	TTTCCCATCAGCATCTTCAACCTG
